# Why Are Healthcare Providers Leaving Their Jobs? A Convergent Mixed-Methods Investigation of Turnover Intention among Canadian Healthcare Providers during the COVID-19 Pandemic

**DOI:** 10.3390/nursrep14030152

**Published:** 2024-08-21

**Authors:** Andrea M. D’Alessandro-Lowe, Andrea Brown, Emily Sullo, Mina Pichtikova, Mauda Karram, James Mirabelli, Randi E. McCabe, Margaret C. McKinnon, Kim Ritchie

**Affiliations:** 1 Department of Psychology, Neuroscience and Behaviour, McMaster University, Hamilton, ON L8S 4L8, Canada; 2 Department of Psychiatry and Behavioural Neurosciences, McMaster University, Hamilton, ON L8S 4L8, Canadamckinno@mcmaster.ca (M.C.M.); 3 Department of Applied Psychology and Human Development, University of Toronto, Toronto, ON M5S 1A1, Canada; 4 Lawrence Bloomberg Faculty of Nursing, University of Toronto, Toronto, ON M5S 1A1, Canada; 5 St. Joseph’s Healthcare Hamilton, Hamilton, ON L8N 4A6, Canada; 6 Homewood Research Institute, Guelph, ON N1E 6K9, Canada; 7 Trent/Fleming School of Nursing, Trent University, Peterborough, ON K9L 0G2, Canada

**Keywords:** turnover intention, job turnover, healthcare, healthcare providers, organizational support, burnout, moral injury, COVID-19 pandemic

## Abstract

Background: Staffing shortages across the healthcare sector pose a threat to the continuity of the Canadian healthcare system in the post-COVID-19 pandemic era. We sought to understand factors associated with turnover intention as well as Canadian healthcare providers’ (HCPs) perspectives and experiences with turnover intention as related to both organizational and professional turnover. Method: A convergent questionnaire mixed-methods design was employed. Descriptive statistics and ordinal logistic regressions were used to analyze quantitative data and ascertain factors associated with turnover intention. Thematic analysis was used to analyze qualitative open-field textbox data and understand HCPs’ perspectives and experiences with turnover intention. Results: Quantitative analyses revealed that 78.6% of HCPs surveyed (N = 398) reported at least a 25% turnover likelihood regarding their organization, with 67.5% reporting at least a 25% turnover likelihood regarding their profession. Whereas regression models revealed the significant impact of years worked, burnout, and organizational support on turnover likelihood for organizations, age, sex, burnout, and organizational support contributed to the likelihood of leaving a profession. Patterns of meaning drawn from participants’ qualitative responses were organized according to the following four themes: (1) Content to stay, (2) Drowning and no one cares, (3) Moral stressors, and (4) Wrestling with the costs and benefits. Conclusions: Many HCPs described weighing the costs and benefits of leaving their organization or profession during the COVID-19 pandemic. Although challenging working conditions, moral stressors, and burnout may play a significant role in HCPs’ experiences of turnover intention, there is ample room to intervene with organizational support.

## 1. Introduction

Staffing shortages across the healthcare sector pose a threat to the continuity of the Canadian healthcare system in the post-COVID-19 pandemic era. Indeed, a report from Statistics Canada [1] revealed 90,000 vacant healthcare positions nationwide in the second quarter of 2023, representing a 9.5% increase in such vacancies since the year prior. Critically, staffing shortages are associated with compromised patient safety, increased patient mortality, increased cost, and elevated psychological burden on remaining staff [2,3,4,5]. Staffing shortages appear to have been exacerbated by the COVID-19 pandemic, as global research points toward elevated turnover intention among healthcare providers (HCPs) during this historic period [6,7]. Turnover intention has been defined as “an individual’s perceived probability of permanently leaving the employing organization in the near future” [8] (p. 3) and has been identified as the best predictor of actual job turnover among nurses [6]. Moreover, turnover intention may refer to leaving one’s organization or profession [6].

Elevated turnover intention among HCPs was reported globally throughout the COVID-19 pandemic. In Canada, while approximately one in four respiratory therapists reported considering leaving their position due to moral distress in the spring of 2021 [9], one in three nurses reported considering leaving their organization, and one in four reported considering leaving their profession entirely, in the latter half of 2020 [10]. Similar reports of turnover intention throughout the COVID-19 pandemic were reported among intensive care nurses in Romania [11], frontline nurses in the Philippines [12], and nurses in Germany [13]. Moreover, almost half of the 124 advanced practice nurses surveyed in the United Kingdom reported considering leaving their job during the initial three months of the COVID-19 pandemic [14], and two in five pharmacists surveyed between December 2020 and January 2021 in Lebanon reported intending to quit their job in the next year [15]. In an integrative review comparing the pre-pandemic literature on turnover among nurses to research produced during the COVID-19 pandemic, Falatah [6] concluded that turnover intention increased substantially during the COVID-19 pandemic, although direct comparisons of turnover intention were difficult to make due to varying definitions and measurement of turnover intention. Similarly, cross-sectional findings from nurses in Qatar revealed that nurses reported greater turnover intention during the COVID-19 pandemic as compared to their retrospective reports of turnover intention before the pandemic period [16].

Elevated turnover intention among HCPs during the COVID-19 pandemic may be partially explained by the widespread increase in moral stressors that HCPs faced during the pandemic period. For example, HCPs described working with a shortage of adequate personal protective equipment, working understaffed with increased workloads, witnessing patients die alone, having to provide patient care that appeared futile, having to remove potentially life-saving resources from one patient to aid a patient with better odds of survival, potentially exposing loved ones to the virus, a perceived lack of support from organizations and governments, and having to take on clinical responsibilities outside of one’s scope of practice due to staffing issues and high caseloads [17,18,19,20,21,22]. In this context, HCPs appeared to be at elevated risk for moral distress and/or moral injury during the pandemic period.

Moral distress was initially conceptualized in the nursing literature to describe the psychological stress faced when prevented from acting in line with professional ethics and values [23]. Moral distress has been associated with negative impacts on patient care, job satisfaction, and attrition, in addition to increased functional impairment and symptoms of post-traumatic stress disorder (PTSD), depression, anxiety, and burnout [9,24,25,26,27]. Historically situated in the military and veteran literature, moral injury is a term initially described as a psychological trauma stemming from a betrayal of what is morally right by an individual with authority in a high-stakes situation [28]. Litz and colleagues [29] later broadened this definition to account for psychological trauma arising when an individual is the agent of a moral transgression rather than the witness or victim. Accordingly, moral injury is often described as psychological, social, behavioral, and spiritual distress and impairment that may take two forms: primary internalizing symptoms (e.g., shame-related outcomes like shame, guilt, social withdrawal) typically presenting when an individual is the agent of the moral transgression, or primary externalizing symptoms (e.g., trust-violation-related outcomes like anger and a cynical worldview) typically presenting when the individual witnesses or is the victim of another individual’s moral transgression [30]. Moral injury may involve a shattered sense of self, decreased connectedness to others, impairing moral emotions and a change in beliefs about the self, others, and the world [30]. Moreover, moral injury has been associated with adverse mental health and functioning, including PTSD, Major Depressive Disorder, substance use, and suicidality [31,32,33,34,35,36,37,38].

Although both moral distress and moral injury share some conceptual properties (e.g., moral emotions in response to a moral stressor), Litz and Kerig [39] contend that moral injury may be a more impactful and impairing expression than moral distress, as moral injury involves incapacitating symptoms cutting across psychological, social, behavioral, and spiritual domains that rupture one’s identity and beliefs. Rosen et al. [40], however, argue that moral distress and moral injury belong on a broader continuum that stretches form moral awareness to burnout. Here, burnout is characterized as “a syndrome of emotional exhaustion, loss of meaning in work, feelings of ineffectiveness, and a tendency to view people as objects rather than as human beings” [41]. In Rosen et al.’s [40] view, burnout represents the ultimate outcome of moral stressors, where the individual is “beyond feeling”, experiencing “an absence of distress or moral investment altogether” [40] (p. 3740). While empirical support for both Litz and Kerig’s [39] and Rosen and colleague’s [40] theoretical continuums have yet to be established, the constructs of moral distress, moral injury, and burnout have each been associated with pandemic-related experiences and increased turnover intention among HCPs [9,11,22,34,42,43,44].

Relatedly, in an investigation of turnover intention related to moral stressors during the pandemic period, Nazarov et al. [22] reported that nearly half (44%) of the 1204 Canadian HCPs surveyed were considering leaving their jobs due to moral distress. Furthermore, statistical modeling pointed toward burnout, moral distress, and trust-violation-related moral injury as significant predictors of job turnover [22]. Here, burnout was the sole mental health variable among PTSD, depression, and anxiety, significantly related to turnover intention [22].

Notably, the literature on turnover intention among HCPs during the COVID-19 pandemic is largely composed of quantitative studies. As such, several factors have been identified as critical for predicting turnover intention, including fear of exposure to the virus, psychological responses to stress, sociodemographic characteristics, adverse working conditions, and organizational support [6,7]. Specifically, whereas fear of contracting the virus [10,45,46], depression [11,13], anxiety [11,47], burnout [42,43], moral distress or injury [9,11,34,42,44], adverse working conditions [48,49], and increases in workload [48] were associated with increased turnover intention among HCPs, resilience [15], social support [12,47,48], and organizational trust or support [13,43,50,51] have been associated with decreased turnover intention among HCPs throughout COVID-19 [7].

Although the extant research raises global concern about elevated turnover intention among HCPs and highlights critical predictive factors, a comprehensive understanding of turnover intention among Canadian HCPs during the COVID-19 pandemic remains absent in the literature. Indeed, most of the research on turnover intention among HCPs during the pandemic used quantitative designs [7], which alone are insufficient for capturing HCPs’ perspectives on and experiences with turnover intention during this historic period. Without a comprehensive understanding of turnover intention among Canadian HCPs throughout the COVID-19 pandemic, the continuity of the Canadian healthcare system in the post-COVID era remains at risk. Accordingly, this mixed-methods study aims to understand turnover intention among Canadian HCPs during the COVID-19 pandemic.

A convergent questionnaire mixed-methods design [52] was used, such that both quantitative and qualitative data were collected from one group of participants via an online questionnaire and analyzed separately, then merged for interpretation. In this approach, quantitative survey data were used to answer the following research questions: (1) What was the prevalence of turnover intention among Canadian HCPs during the COVID-19 pandemic? and (2) What factors were associated with turnover intention? Based on the extant literature reviewed above, socio-demographic factors (i.e., age, sex, years worked), exposure to morally distressing events and experiences of moral injury (i.e., shame- and trust-violation-related moral injury), mental health impacts (e.g., burnout, PTSD, depression, anxiety) and resilience and supports (i.e., resilience, social support, organizational support) were considered in the quantitative portion of this study. In line with the available literature, we hypothesized that increased turnover intention would be significantly associated with female sex, increased age, exposure to morally distressing events, and increased reports of moral injury, burnout, post-traumatic stress, depression, and anxiety. We further hypothesized that decreased turnover intention would be significantly associated with higher resilience, social support, and organizational support.

At the same time, qualitative data collected via optional open-field text boxes on the online questionnaire were analyzed to answer the research question: What were Canadian HCPs’ perspectives and experiences related to their reported turnover intention? Finally, upon merging the data for interpretation, a mixed-methods research question was addressed: To what extent did the quantitative results agree with HCPs qualitative reports on turnover intention? The reason for collecting both quantitative and qualitative data was to converge the two forms of data to yield deeper insight into turnover intention among HCPs than either type of data could produce alone [52]. Additionally, intention to leave one’s healthcare organization and to leave one’s healthcare profession were considered in this study to capture potential nuances associated with turnover in each regard. The results of this investigation yielded information critical for the development of retention programs aimed at sustaining and encouraging a robust workforce and, consequently, reinforcing the continuity of the Canadian healthcare system.

## 2. Methods

### 2.1. Study Design

We used the questionnaire variant of the convergent mixed-methods design [52] to understand turnover intention among HCPs during the COVID-19 pandemic. The purpose of the convergent mixed-methods design is to collect, analyze, and compare both quantitative and qualitative data on a single research topic to allow for a better understanding of the research problem than each type of data can offer alone [52]. Our study specifically made use of the questionnaire variant of this design [52], where both open- and closed-ended questions were included on a single survey administered to HCPs across Canada, yielding both quantitative (i.e., self-report surveys) and qualitative (i.e., open-field textboxes) data.

A pragmatic worldview was assumed throughout the research process, such that we made the following ontological and epistemological assumptions, respectively: reality is both singular and multiple and knowledge is determined practically by the appropriate means to answer research questions [52] (p. 37). The assumptions of the pragmatic lens were used to guide all aspects of the research process.

### 2.2. Procedure

Canadian HCPs were invited to participate in an anonymous online questionnaire comprised of open- and closed-ended items housed on Research Electronic Data Capture Software (REDCap) [53,54]. Eligible participants resided in Canada, were at least 18 years of age, were employed during the COVID-19 pandemic as a HCP, and reported competency in speaking and writing in English. Participants provided electronic informed consent before completing the questionnaire. This study involved data pulled from a broader investigation of HCPs’ mental health and experiences during and after the COVID-19 pandemic that was approved by the Hamilton Integrated Research Ethics Board (#12667). Data for this study were collected between May 2022 and August 2023.

**Figure 1:**
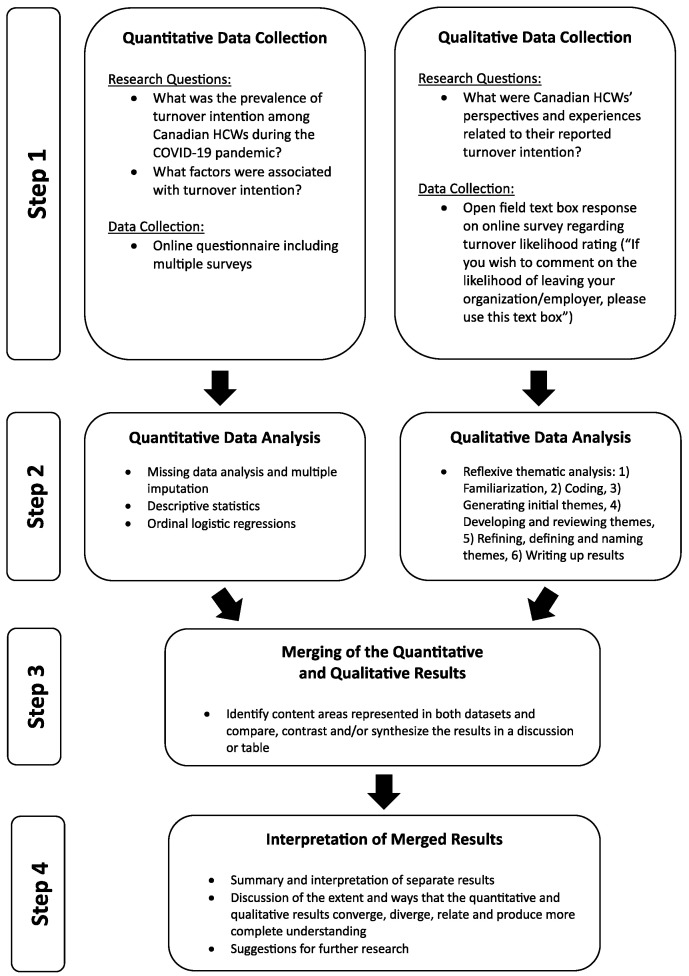
Convergent Questionnaire Mixed-Methods Design Procedural Flowchart. This procedural flowchart depicts the four steps of the convergent mixed-methods design followed in this study as per the guidelines offered by Creswell and Clark [52].

A procedural flowchart based on Creswell and Plano Clark’s [52] guidelines for mixed-methods research is presented in Figure 1. As depicted in the figure, step one of this study involved collecting both quantitative and qualitative data from the same set of HCPs via an online questionnaire. In step two, the quantitative and qualitative data were analyzed concurrently and separately. The quantitative and qualitative results were integrated in step three, whereby the results from both datasets were compared and represented in discussion and in a joint display. Finally, interpretation of the convergence and divergence of the quantitative and qualitative data was completed in step four to answer the mixed-methods research question.

### 2.3. Quantitative Data Collection and Analysis

The online questionnaire included several closed-ended questions, including survey measures capturing demographic information, turnover intention, moral distress, moral injury, burnout, PTSD, depression, anxiety, resilience, and social and organizational support. These variables were selected from the broader study from which these data were drawn based on the relevant literature on turnover among HCPs during the pandemic, reviewed above. Details regarding the survey instruments used for quantitative data collection are presented in Table 1.

#### Data Preparation and Analysis

A total of 398 (N = 398) data entries were received in the data collection period. Missing data were analyzed and imputed using the multiple imputation procedure available on Statistical Software for Social Sciences (SPSS, Version 29) [62]. Specifically, multiple imputation with 10 imputed datasets was employed using a fully conditional specification and predictive mean matching for scale variables. Data analysis was completed on SPSS [62]. Descriptive statistics were run to characterize the sample. The unimputed dataset was used to calculate descriptive statistics. A series of simple ordinal linear regressions were constructed to assess the relationships among each of the independent variables and both intentions to leave an organization and profession, respectively. Based on the simple regressions, ordinal linear regressions were constructed to assess the probability of turnover intention for both an organization and profession, respectively. Regression assumptions were tested on all imputation iterations and screened for major discrepancies. Parameter coefficients in the ordinal logistic regressions were calculated using the pooled estimate of the multiply imputed datasets, as per SPSS’s imputed data analysis functions. An alpha level of *p* = 0.05 was used for all analyses.

### 2.4. Qualitative Data Collection and Analysis

Upon rating their current turnover intention for both their organization and profession, participants were invited to comment on their ratings in an open-field textbox on the questionnaire. The question read: “If you wish to comment on the likelihood of leaving your organization/employer, please use this text box”, and the same wording was used regarding leaving one’s profession. Seven qualitative data excerpts were received in French and translated to English via Google Translate and checked by a native French speaker on the research team. Qualitative data excerpts were analyzed using qualitative thematic analysis [63] on MAXQDA 2024 Software [64]. This analysis technique focuses on identifying patterns of meaning across the dataset. The thematic analysis involved the following steps: data familiarization, coding, generating, developing, and revising themes to capture patterns of meaning across the data [63].

Some HCPs provided one-word responses regarding retirement or that they had already left their position or profession without offering further details. Others mentioned they were searching for a job in an alternative sector (e.g., teaching English) without providing insight into the context of their occupational shift. These qualitative responses were excluded from the analysis.

The initial phases of qualitative data analysis were grouped according to organizational turnover, professional turnover, and turnover likelihood rating. However, there were no distinct patterns of meaning perceived between qualitative responses pertaining to organizational turnover and professional turnover. Furthermore, distinct patterns of meaning were not perceived across the levels of turnover likelihood. As such, the qualitative data were analyzed as a whole rather than based on subgroups of organizational turnover, professional turnover, or turnover likelihood rating. Participant numbers are presented in parentheses following qualitative data excerpts presented in the results section.

## 3. Results

### 3.1. Quantitative Data

#### 3.1.1. Sample

Participant demographic information (N = 398) is presented in Table 2. Most participants were female (91.5%), nurses (56.5%), and resided in Ontario (62.3%). The average age of participants was 42.17 (SD = 11.71), and the average years worked in the healthcare field was 13.80 (SD = 11.14). Descriptive statistics for measures included in modeling are presented in Table 3.

#### 3.1.2. Turnover Intention—Organization

Simple Regressions. A series of simple cumulative odds ordinal logistic regressions with proportional odds were performed to ascertain the effects of sex, age, years worked, perceived organizational support, social support, resilience, moral distress, moral injury (shame- and trust-related separately), burnout, PTSD, depression, and anxiety on the odds of expressing a higher organizational turnover intention, independently (Table 4). Results from the simple regressions were used to determine the variables to be included in the next stage of modeling. Organizational support was significantly associated with decreased odds of expressing a higher turnover intention (*p* < 0.05). Years worked, moral distress, shame-related moral injury, trust-violation-related moral injury, burnout, post-traumatic stress, depression, and anxiety were each independently significantly associated with increased odds of expressing a higher turnover intention (*p* < 0.05). Sex, age, sex^×^age, social support, and resilience were each not significantly associated with a change in the odds of expressing a higher turnover intention (*p* > 0.05).

Full Model. Cumulative odds ordinal logistic regressions with proportional odds were performed to ascertain the effects of years worked, moral distress, moral injury (shame- and trust-violation-related), burnout, PTSD, depression, anxiety, and perceived organizational support on the odds of expressing a high organizational turnover intention. Regressions were split into four segments, organizing variables into (i) occupational factors, (ii) exposure to morally distressing events and outcomes of moral injury, (iii) mental health impacts, and (iv) supports to assess the change in variance explained with the addition of each set of variables. Parameter estimates for each iteration of the model-building process are presented in Table 5. Notably, although years worked, moral distress, and trust-violation-related moral injury explained a large proportion of the variance in turnover intention in the second iteration of the model, these effects were washed out when burnout was added as a predictor with other mental health variables in the third model iteration. Moreover, of the various mental health impacts considered in the third iteration of the model (i.e., burnout, PTSD, depression, and anxiety), burnout alone returned as significantly associated with higher odds of turnover. Finally, perceived organizational support was added in the fourth model and was significantly associated with decreased odds of a higher turnover likelihood.

The final model was determined by the preceding model-building steps. This model is the most parsimonious and includes years worked, burnout, and organizational support (Table 6). The final model accounted for approximately 34.9% (Nagelkerke *R*^2^ = 0.349) of the variance in turnover intention. The assumption of multicollinearity among independent variables was assessed and met (VIFs < 2). There were proportional odds, as assessed by a full likelihood ratio test comparing the fitted model to a model with varying location parameters, χ^2^(6) = 12.344, *p* = 0.055. The deviance goodness-of-fit test indicated that the model was a good fit to the observed data, χ^2^(1477) = 1022.08, *p* = 1.00, but most cells were sparse with zero frequencies in 80% of cells. However, the final model statistically significantly predicted the dependent variable over and above the intercept-only model, χ^2^(3) = 151.26, *p* < 0.001. Parameter estimates revealed a significant increase in the odds of the likelihood of leaving one’s occupation with increased years working and burnout and a significant decrease in the odds of turnover likelihood with increased perceived organizational support (Table 6).

#### 3.1.3. Turnover Intention—Profession

Simple Regressions. A series of simple cumulative odds ordinal logistic regressions with proportional odds were performed to ascertain the effects of sex, age, years worked, moral distress, moral injury (shame- and trust-violation), burnout, post-traumatic stress, depression, anxiety, resilience, social support and perceived organizational support on the odds of expressing a high professional turnover intention, independently (Table 7). Results from the simple regressions were used to determine the variables taken to the next stage of modeling. Age was significantly associated with increased odds of higher turnover intention, especially among females (*p* < 0.05). Whereas increased years worked, moral distress, moral injury (shame-related and trust-violation), burnout, post-traumatic stress, depression, and anxiety were independently associated with increased odds of higher turnover likelihood, organizational support was significantly associated with decreased odds of expressing a higher turnover intention (*p* < 0.05). Sex, social support, and resilience were each not significantly associated with a change in the odds of expressing a higher turnover intention (*p* > 0.05).

Full Model. Cumulative odds ordinal logistic regressions with proportional odds were performed to ascertain the effects of sex^×^age, moral distress, moral injury (shame- and trust-violation-related), burnout, PTSD, depression, anxiety, and perceived organizational support on the odds of expressing a high organizational turnover intention (Table 8). To reduce redundancy, years worked was dropped and age was retained based on the significant sex^×^age interaction supported by the simple regressions. Regressions were split into four segments, organizing variables into (i) demographic factors, (ii) exposure to moral distressing events and experiencing moral injury, (iii) mental health impacts, and (iv) support to assess the change in variance explained with the addition of each set of variables. Parameter estimates for each iteration of the model, along with the final model, are presented in Table 8. Notably, the pattern of results was similar to what was found for organizational turnover, where the effects of moral distress, and trust-violation-related moral injury were washed out when burnout was added to the model.

The final model was determined by the preceding model-building process. This model is the most parsimonious and includes sex^×^age, burnout, and organizational support (Table 9). The final model accounted for approximately 31.9% (Nagelkerke *R*^2^ = 0.319) of the variance in turnover intention. The assumption of multicollinearity among independent variables was assessed and met (VIFs < 2). There were proportional odds, as assessed by a full likelihood ratio test comparing the fitted model to a model with varying location parameters, χ^2^(12) = 12.44, *p* = 0.411. The deviance goodness-of-fit test indicated that the model was a good fit to the observed data, χ^2^(1456) = 951.75, *p* = 1.00, but most cells were sparse with zero frequencies in 80% of cells. However, the final model statistically significantly predicted the dependent variable over and above the intercept-only model, χ^2^(4) = 131.95, *p* < 0.001. Parameter estimates revealed that an increase in age was associated with a significant increase in odds of expressing a high turnover intention among females only. Whereas higher burnout was associated with an increase in the odds of turnover intention, higher perceived organizational support was significantly associated with decreased odds of turnover intention (Table 9).

### 3.2. Qualitative Data

**Figure 2:**
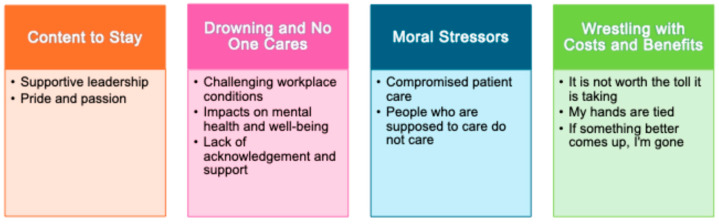
Themes and subthemes capturing patterns of meaning across HCPs’ qualitative text box responses regarding their turnover likelihood ratings. Final themes and subthemes capturing patterns of meaning across HCPs’ qualitative text box responses regarding their turnover likelihood ratings. Four themes and ten subthemes resulted from the thematic qualitative analysis.

When invited to comment on their turnover likelihood ratings regarding both their organization and profession, some HCPs described being content to stay due to their pride and passion for the job, coupled with supportive leadership. Most HCPs, however, focused their comments on challenging working conditions created by low staffing levels and high workloads that, in turn, impacted their mental health and resulted in patient safety being compromised. HCPs also described a perception that leaders at multiple levels did not acknowledge the incredulous circumstances HCPs worked in during the pandemic, nor did they offer adequate support. Here, HCPs described a sense of betrayal where the people they believed were supposed to care for them did not. Many HCPs described wrestling with the thought of leaving their organization or profession. For some, the toll of the working circumstances appeared to outweigh any benefit of remaining in their occupation or profession. Others had a strong desire to leave yet felt constrained from leaving due to practical or personal reasons. Finally, many HCPs de-scribed being on the lookout for a better employment option, such that they were ready to make the move if something else came up. Patterns of meaning drawn from participant responses are organized according to the following four themes: (1) Content to stay, (2) Drowning and no one cares, (3) Moral stressors, and (4) Wrestling with the costs and benefits (Figure 2).

#### 3.2.1. Content to Stay

About a dozen textbox responses depicted HCPs as content to remain in their current occupation or profession. The positive impacts of supportive leadership were evident, along with a sense of pride in one’s work and passion for the caring profession.

#### 3.2.2. Supportive Leadership

HCPs described the positive impact of supportive leadership on their work experiences during the pandemic: “I feel secure and safe with my current employer and location” (P86). Notably, HCPs describing a contentment to stay were not exempt from the challenging pandemic circumstances described by others. Rather, support from leadership appeared to give HCPs a sense of safety in remaining in their position despite the challenging workplace circumstances: “I have struggled during the pandemic, but my employer has been overwhelmingly supportive, and a positive factor in remaining in my current role and profession” (P191). This was echoed by another HCP who described that they may have chosen to leave given the pandemic circumstances had it not been for the support from leadership and others in the workplace: “If it were not for the people I work with and the values of the family that own the organization, I may very well have decided that the strain under COVID was too much and left” (P73).

#### 3.2.3. Pride and Passion

HCPs who described being content to stay in their occupation or profession highlighted the pride they hold for the work that they do and the passion with which they serve their patients. For example, HCPs commented: “I love what I do and the population I work with” (P230), “I still enjoy my work” (P198), and “…I also love caring for others” (P731).

HCPs working in spiritual care described a sense of honor related to being able to serve their patients, especially in the difficult circumstances of the pandemic: “I feel fortunate to have a full-time job as a spiritual care/soul care provider” (P84). This was echoed by another spiritual care provider who wrote: “I think it is more important than ever that I maintain my job in this (post-) pandemic situation. Spiritual care of patients, loved ones, AND STAFF is more important now than ever” (P136, emphasis original).

The pride and passion with which these HCPs described living out their occupations appeared to be a driving factor, rendering leaving one’s position or profession of no interest. As one HCP said: “Never! As long as I have my health, I will spend my life doing what I love!” (P181).

#### 3.2.4. Drowning and No One Cares

HCPs described challenging working conditions created by chronic staffing shortages and increased workloads. Working in these conditions, in turn, negatively impacted HCPs’ mental health and well-being. Moreover, HCPs described the perception that organizational and professional leadership, as well as the government, failed to adequately acknowledge the strain HCPs were under and failed to provide sufficient support.

#### 3.2.5. Challenging Workplace Conditions

HCPs described staffing shortages as a daily issue contributing to the development of challenging workplace conditions (e.g., “rest from stress, short staffed every day” (P83), and “always understaffed and under-supplied” P94). This was echoed by another who offered: “feel unsupported, severely understaffed. Sinking burning ship” (P384). Indeed, for a HCP in a managerial role, staffing became a predominant part of their job during the pandemic: “Staffing has become 85% of my job, which is not something I want to do” (P58).

HCPs highlighted that staffing issues were a major problem in their workplace that, in turn, created unrealistically high workloads. For example, one participant described their workload after being deployed to a new department when many staff members left the organization as dangerous and stressful: “… There was an exodus from the department, and [they] were doing forced re-deployments. The shift that I took over from had 13 acute patients for 1 [registered nurse] and 1 third-year nursing student. Dangerous and stressful” (P337). High workloads resulted in HCPs being unable to meet the expectations of management: “I love my job but cannot meet the expectations of management”( P180) and patients: “Patients are more entitled than ever, and it is very exhausting to try to meet all of their expectations” (P363) during the pandemic.

The demands of the workplace contributed to the perception of a “toxic” workplace environment (e.g., “Psychological harassment & toxic workplace” P255) that further heightened adverse working conditions. One HCP highlighted that vacation denial related to staffing shortages was particularly relevant to their likelihood to leave: “No vacation because of same, forced to do work you shouldn’t because of ratios” (P115). Indeed, another HCP stated, “Unapproved vacation, stigma about using sick time, never staffed properly, stupid ideas about ‘team nursing’” (P352), suggesting a workplace culture in which staff are discouraged and even prevented from taking time off. Notably, in this nurse’s comment is a sense of cynicism regarding the idea that staff can work together and adequately complete their duties despite a workplace environment where staff are prevented and discouraged from taking time off and are always working short-staffed.

#### 3.2.6. Impacts on Mental Health and Well-Being

The impact of challenging working conditions on HCPs’ mental health and well-being was highlighted by many HCPs. For example, a nurse who stated: “Too hard now can’t always work short anymore not worth it, mentally, emotionally, and financially” (P1144), demonstrating the personal toll of chronic staffing issues. Another HCP highlighted that staffing issues and refused time off have taken a toll on their mental health: “The stress is a huge toll on my mental and physical [well] being and I’m unsure if I’ll be able to keep up with it if things stay as they currently are in regard to staffing, allowing for time off/holidays and manage [patient] loads” (P247).

An HCP working in a pharmacy setting noted that the job was inherently stressful, but the pandemic added compacting layers of stress: “Stressful job to start but COVID-19 pushed pharmacies to the edge” (P157). Moreover, HCPs listed burnout as related to their turnover ratings (e.g., “burned out from nursing” P309). Burnout led some to take early retirement, with an undecided future on returning casually: “I am completely burned out. I am planning to take early retirement, just figuring out timing. Whether I come back later as a casual is not decided” (P102). Notably, some HCPs connected the mental health toll of the job to a toxic environment: “Our management and toxic work environment has caused me to develop PTSD and has caused massive emotional trauma” (P1215).

HCPs described losing the positive regard they previously held for their jobs, such that they “don’t have much joy anymore” (P1264) in their work and “no longer enjoy my job” (P372). Depression and exhaustion were additionally described relating to staffing issues, having to wear masks for long periods, uncertainty about contracting the virus, isolation, loss of wages, chronic short staff, violations against best practice and working in situations that pose a risk to licensing: “Exhaustion from chronic short staffed leaving unsafe staffing ratios” (P115) and “Wearing masks for entire shifts, not knowing if I will become infected for a 3rd time has me rethinking working in healthcare 10 days isolation with loss of wages as become very depressing” (P64). This HCPs’ account demonstrates the compacting nature of many workplace factors that ultimately brought negative psychological and physical impacts to staff.

#### 3.2.7. Lack of Acknowledgment and Support

A perceived lack of adequate acknowledgment and response to the working conditions by management and leadership resulted in HCPs “Feeling devalued & unsafe” (P272). Some perceived that management denied issues altogether: “Inadequate supports, poor working conditions, inadequate staffing to provide safe care to patients, management denying current crisis” (P177). Expanding on this experience, other HCPs described that management failed to recognize the challenging working conditions that staff faced daily, leading staff to feel disposable and considering alternative employment: “I wonder if other nursing jobs any better, gone are days of doing the job you were hired for, seems we are disposable, can be used and moved where ever people want us, without added support, monies, etc.” (P343).

One nurse perceived that management failed to advocate for nursing staff concerning legislation during the pandemic period: “Bill 124 and the lack of management to recognize and to advocate for its nursing staff. This is hospitals across all of [province]. I have not heard even one leadership team speak to it” (P163). Some HCPs perceived that the government did not support healthcare during the pandemic period: “I already left my job in 2021 and still don’t think I can work in the profession any longer after how awful we are treated in healthcare and the fact our government could care less” (P299).

One HCP described the state of the healthcare system as at “war”, highlighting a perceived disconnect between those making decisions and those on the frontlines: “There is a healthcare war at the moment business world vs common sense folks living in the real world not a fantasy world stuck to a computer” (P203). Relatedly, a nurse reported a sense of disenchantment with the profession, such that they are “eager” to leave since learning how “the business” operates: “I was a RN in my home country and have been working towards getting my license in [province], for me this was always a temporary job, as my workplace does not have any RN positions. For a while, I considered staying but after seeing [how] the business is run, I am eager to leave whenever I get the first chance” (P34).

#### 3.2.8. Moral Stressors

The working conditions HCPs faced during the pandemic period appeared to create situations that transgressed HCPs’ deeply held values. Specifically, whereas chronic staffing shortages and increased workloads resulted in compromised patient care, the perceived lack of acknowledgment and support from leaders whom HCPs viewed as having the responsibility to care for their health and well-being resulted in a sense of betrayal.

#### 3.2.9. Compromised Patient Care

Across HCPs’ excerpts was a sense that the workplace conditions (e.g., increased workload, staffing shortages) compromised patient safety and patient care. Compromised care directly contradicts HCPs’ moral imperative as professionals with a duty to do no harm and provide adequate care. As one HCP said: “[I] signed up to be a nurse to care for people, high [expectation] of job to meet demands/expectation unreasonable. Caseloads [too] high, not enough [time] to care for individuals with quality care” (P182). Participating in care that is not in patients’ best interests was described as a reason why one nurse will no longer follow through with her “dream” career: “I have become a NP with school and will no longer be a RN. This has always been my dream, but it was even more amplified during the pandemic–I hated being understaffed and not being able to provide THE BEST CARE” (P352, emphasis original). Some HCPs reported working with poorly trained team members or having to care for patients for which they did not have adequate training to care for: “During the pandemic demands on nursing increased significantly. We worked short-staffed almost every shift, were required to take care of patients that were not specialized for our unit and workload increased exponentially. Burn out was significant and considered leaving nursing due to treatment and nursing conditions” (P131).

Witnessing other professionals dictate care that was not in line with standards of practice was also described by HCPs in their comments on turnover likelihood: “…Tired of things not changing and the doctors dictating care that isn’t best practice” (P115). Furthermore, the pandemic conditions resulted in a shift in the way healthcare was offered, such that some HCPs perceived that patients who did not test positive for COVID-19 were neglected from services: “…Extremely restricted access to health care during the pandemic… essentially if you don’t have COVID, you don’t qualify for urgent, essential, or routine health services” (P101).

Finally, a HCP perceived that working in the organization would prevent her from living out her professional and personal code of ethics: “I do not believe it safe to work at those organizations and maintain my professional/personal code of ethics or uphold my knowledge both academically and experientially” (P375).

#### 3.2.10. People Who Are Supposed to Care Do Not Care

Embedded within HCPs’ perceptions that leaders at multiple levels failed to acknowledge and adequately support staff during the pandemic was the notion of betrayal at the hands of individuals with a responsibility to care for HCPs, namely organizational and professional leaders and the government. Indeed, reflecting on their turnover likelihood, HCPs definitively described: “There is no support from management, and they just simply do not support the frontline staff nor listen to our concerns” (P354). Another HCP described being ignored by leadership when raising concerns for patient safety due to the staffing issues and workload: “I brought forward patients’ safety, professional practice, and personal safety issues at 4 organizations and was [dismissed], devalued, [disregarded]” (P375).

HCPs described the perception that structures intended to protect the public and advocate for safe patient ratios were not adequately responding to the staffing crisis nor appropriately compensating staff, thus placing HCPs in a position to work in a profession that was not acting in line with its mandate to protect the sick while feeling under-compensated: “The unsafe patient to staffing ratios have [worsened] and the lack of appropriate compensation for working various specialties is ridiculous. The College of Nurses is the governing body that is meant to protect the public. But they are refusing to implement appropriate nurse to patient ratios thus putting the public at risk. Why work in a profession that is meant to care for the sick when the very governing body that is out there to protect the public isn’t even implementing the appropriate standards to protect the public. By not identifying safe and appropriate nurse to patient ratios it prevents nurses from refusing unsafe work, thus the cause of the mass exodus” (P256).

Relatedly, a social worker echoed this call for their profession to advocate on behalf of its workers: “The social work profession has done zero to advocate for social justice regarding mandates and carceral logics that have been implemented in long term care homes. Social work is supposed to advocate for residents and families not enslave them into social isolation and solitary confinement. I am absolutely appalled at the entire medical field” (P19).

#### 3.2.11. Wrestling with the Costs and Benefits

Regardless of their turnover intention rating with respect to one’s organization or profession, HCPs described wrestling with the costs and benefits of leaving and appeared to be at different steps in this process. Some HCPs did not desire to leave their organization or profession, per se, but noticed that the costs of remaining in their job currently outweigh the benefits of the role, making remaining in the position unsustainable. For others, they appeared beyond acknowledging that the costs of the job outweigh its benefits and were at a place where they would like to leave but were prevented from doing so for pragmatic reasons. Finally, some HCPs described being ready to move into a new role if something better arises, suggesting that when the right opportunity presents itself, they will move swiftly to take advantage of it. Although numerous HCPs found themselves feeling like their professional work was not worth the toll it was taking or wanting to leave but feeling stuck, HCPs nonetheless appeared to be at different stages of a negotiation process in their decision-making, demonstrating that turnover intention is a complex process that often does not have a clear outcome.

#### 3.2.12. It Is Not Worth the Toll It Is Taking

In describing a process of weighing the costs and benefits of staying or leaving their occupation or profession, many HCPs emphasized that the costs of remaining in the profession presently outweighed any benefits, resulting in a sense that remaining in the profession was not worth it.

The mental health and well-being impacts of working through challenging conditions are one of the significant “costs” to remaining in one’s position, especially in the context of inadequate support. HCPs described: “I am far from home and feel like the lack of resources and supports are getting to be too much and is stress of the job is not worth the toll it’s taking” (P247). HCPs expect the job to come with strain, but they can only take so much. When the impacts of the job reach a tipping point where HCPs do not see a feasible way to continue their job and care for themselves, they may find themselves in the position of believing the benefits of the job are not worth the toll it is taking. Indeed, HCPs noted: “It depends on when the work becomes too mentally/emotionally taxing without enough benefit to keep me then I will find something else” (P611) and “If my current duties expand without adequate supports or benefits, or things become more stressful and hit a breaking point, I will leave” (P109). Perceiving the mental health toll of their work as a significant cost resulted in some HCPs thinking about retiring early, dropping down to part-time hours, or leaving the profession entirely:

“If something doesn’t change, I’m leaving the industry to go where I am valued and not worked to death” (P262).

“I can retire in about a year and if work life balance does not improve, which I’m hoping it will, I will seriously consider retiring early” (P206)

“I’d like to be part time, I am just returning from a stress leave and being in the emergency department or maybe just healthcare in general seems unsustainable” (P353).

“I have experienced significant mental and physical health problems exacerbated by my role, and I don’t know how much longer I can continue in this job. I may leave the profession” (P370).

HCPs further described weighing the costs and benefits of remaining in the position long-term and having a bleak outlook on the long-term impacts of remaining in the profession: “I don’t think it’s a sustainable career to do for 30 years. Not sure what else I want to do though…” (P251).

Another major “cost” to remaining in one’s organization or profession described across the dataset was the notion that “health care does not pay” (P71). Respondents described a sense that there is “not enough pay for the stress and strain of our daily working situation” (P301) throughout the dataset. A nurse stated that their profession as a whole is underpaid and connected this to being under appreciated: “I am an RPN, and our profession is under paid and underappreciated” (P334). At the organizational level, some HCPs noted that their organization paid less compared to other similar settings: “The rate of pay is below other retirement homes” (P51). A HCP in a managerial position also commented on the perceived lack of financial compensation, especially when compared to their team members: “Inadequate compensation, getting paid same or less than some of the team members I manage” (P58). Finally, one HCP reported leaving their current position due to “poor pay and poor conditions” after already recently leaving a position: “Previously left hospital position during COVID: poor working conditions and toxic manager that became worse. Working in community. Poor pay [and poor] conditions already leaving for a different position” (P20).

As such, the perception of being underpaid for their work appears to impact some HCPs who leaned toward leaving given that they believed the toll of the job was not worth it when their pay was not adequate.

#### 3.2.13. My Hands Are Tied

Other HCPs appeared to already have a clear sense that they did not want to remain with their organization or in their profession but felt constrained from acting on their desire to leave for pragmatic reasons. Notably, the desire to leave appears directly related to the challenging working conditions in the healthcare sector, as described above: “I wish I could retire today as I am unhappy with the current situation in healthcare” (P278). The tension between wanting to leave and feeling constrained from leaving demonstrates the push and pull of weighing the costs and benefits of remaining in the job: “When you work for municipal government (public health), it’s the golden handcuffs…even if things really frustrate you about your employer, your salary, benefits, vacation and union protection (if unionized) are too good to leave” (P190).

Losing out on retirement benefits was mentioned by many HCPs as a reason why they have not acted on their desire to leave their profession: “Would lose retirement benefits by leaving” (P123). Indeed, some explicitly stated that although they wish they could leave, they will not leave until their retirement package has been sorted out: “Would like to retire if given decent package, considering going to another employer if has same pension plan, too close to retirement to just leave otherwise (wish I could though)” (P366). Among excerpts noting retirement as the reason not to leave was a sense of holding out for retirement, as if retirement is the light at the end of the tunnel that motivates HCPs to press on despite the current state of the working conditions: “5 years until I can draw pension. Trying to last until then” (P164). Moreover, although some described holding out for retirement, they were clear in their discouragement for others to join the profession: “Too close to retirement to [change] careers now. But I would not encourage anyone to become a nurse” (P192).

Whether close to retirement or not, HCPs described leaving as a financially unwise decision that prevents them from acting on their desire to leave, with many stating: “can’t afford to leave the job” (P303). HCPs noted their personal responsibilities to care for their families as reasons their hands are tied when it comes to leaving their organization or profession: “Financially I cannot leave my profession. I have a young family to support and their future to plan for. If I were less than 5 years into my nursing career, I would be 50% likely to leave” (P237). HCPs noted that their current pay is not sufficient given the current cost of living: “Salary is insufficient. We aren’t receiving cost of living increases. I can no longer pay bills. Less than 10% raise in 10 years while coast of living has gone up approx. 40%” (P372). HCPs described that if leaving were financially viable, they would do so given the present working conditions: “If I could afford to leave, I would go now. My job is getting very hard, and I don’t think it will get any easier any time soon. I used to love it” (P222).

Other HCPs described a sense that leaving would be dissonant with all that they have sacrificed to obtain their current role. Some noted that they have “spent too much money and time to change now” (P371), while others shared that “I have worked too long for my seniority, benefits and vacation to leave but I have considered it” (P192). Similarly, one HCP said: “Been there too long to leave but would if pension was not affected and there were jobs that paid as well. Would love to be able to leave healthcare. [Love] my job but it has burnt me out” (P287). Others have not acted on their desire to leave their organization or profession simply due to lack of opportunity: “this is the only hospital to work at” (P341), often in remote areas: “Living in a rural area, there are limited opportunities to work locally within an acute care setting” (P237).

#### 3.2.14. If Something Better Comes Up, I Am Gone

Some HCPs appeared to be waiting for the right opportunity to arise to signal their departure. As one HCP said: “If I leave my employer, I will be leaving the field of nursing altogether. I’m not quite there yet/the right opportunity hasn’t come my way” (P281). These folks appear to be actively on the lookout for better opportunities where the benefits of such a position outweigh the costs: “I’m still undecided. But if an offer comes along, I will leave. Every day or every 2 days I look at websites such as Indeed, Career, Beacon… Sometimes I convince myself that I only have 7–8 years of work left before I retire, to make myself comply and endure my work” (PF18).

Most comments in this regard were “if, then” statements where HCPs noted that “if” an opportunity arose that offered greater benefits (e.g., setting, compensation, location), “then” they will leave. For example:

“If a better job came up in a private setting, then yes, I would. With the hope of being valued” (P199).

“If a comparable role and compensation was available outside of LTC I would consider a change” (P20).

“If I get a job closer to home in my field I will consider” (P21).

“I would leave immediately if I could find something comparable in terms of job security, pension, benefits and wage” (P279).

The notion of awaiting the right opportunity appeared directly linked to the working conditions described above. HCPs noted that they are sure to leave if a well-paying position opened up: “Again if I could find a job that pays me well uses my knowledge [I] would actively leave. Years and years of abuse by public management have taken [their] toll. The pandemic was just icing on the cake” (P263).

### 3.3. Merged Results

A joint display of the merged quantitative and qualitative results is presented in Table 8. As described in Table 10, the qualitative findings converged with and expanded upon the results of the quantitative phase.

## 4. Discussion

The purpose of this study was to gain a comprehensive understanding of turnover intention among Canadian HCPs during the COVID-19 pandemic via qualitative and quantitative methods. In this discussion, we summarize the quantitative and qualitative findings independently, compare our findings to the broader literature, and interpret the merged results.

### 4.1. Summary of Quantitative Results

The research questions driving the quantitative strand of our study were: (1) What was the prevalence of turnover intention among Canadian HCPs during the COVID-19 pandemic? and (2) What factors were associated with turnover intention? Our quantitative results demonstrate that 78.6% of HCPs surveyed between May 2022 and August 2023 reported being at least 25% likely to leave their organization. Moreover, 67.5% of the HCPs reported being at least 25% likely to leave the healthcare profession entirely. Our regression modeling for organizational turnover intention revealed that although years worked, moral distress and trust-violation-related moral injury explained a large proportion of the variance in turnover intention in the second iteration of the model, these effects were washed out when burnout was added as a predictor with other mental health variables in the third model iteration. Indeed, of the various mental health impacts considered in the third iteration of the model (i.e., burnout, PTSD, depression, and anxiety), burnout alone returned as significantly associated with higher odds of turnover. Moreover, perceived organizational support was added in the fourth iteration of the model and was significantly associated with decreased odds of a higher turnover likelihood. Thus, the final model pointed toward years worked and burnout as significantly associated with an increased turnover likelihood, while orgnaizational support was significantly associated with decreased turnover likelhood. The results of the regressions pertaining to turnover intention regarding one’s profession were identical to those of the models constructed for organizational turnover, save for the importance of the age-by-sex interaction, demonstrating that increased age was significantly associated with a higher turnover likelihood for females. The quantitative results of this investigation are largely consistent with other reports of turnover intention prevalence and associated factors during the COVID-19 pandemic [6,7,9,11,13,34,42,43,50,51].

### 4.2. Summary of Qualitative Findings

The research question driving the qualitative arm of this study was: What were Canadian HCPs’ perspectives or experiences related to their reported turnover intention? Our qualitative findings indicate that some HCPs were content to stay in their jobs, a state often related to supportive leadership and pride and passion for their work. Most qualitative excerpts, however, described HCPs’ perceptions of challenging working conditions created by low staffing levels and high workloads that, in turn, impacted HCPs’ mental health and well-being. Moreover, HCPs perceived that leaders at multiple levels did not acknowledge the incredulous circumstances HCPs’ worked in during the pandemic, nor did they offer adequate support. The transgression of HCPs’ moral values was embedded in these accounts such that HCPs perceived the workplace context to compromise patient care and described a sense of betrayal when people they believed were supposed to care for them did not adequately demonstrate that care. Critically, many HCPs described wrestling with leaving their organization or profession in some capacity. Some HCPs indicated that the toll of the working circumstances outweighed any benefit of remaining in their occupation or profession. Others had a stronger desire to leave yet felt constrained from leaving due to practical or personal reasons. Finally, some HCPs described being on the lookout for a better employment option, such that they were ready to make the move if something else came up. HCPs’ open-field textbox descriptions of challenging workloads, compromised care, and a perceived lack of support from various levels of leadership are consistent with accounts of challenges and moral stressors faced by HCPs worldwide during the COVID-19 pandemic [18,19,20,21].

### 4.3. Interpretation of Merged Results

As depicted in Table 10, the quantitative and qualitative results of this investigation appear to converge and offer expanded insight into turnover intention among HCPs during the COVID-19 pandemic. Concerning the prevalence of turnover intention, the quantitative results demonstrated that a substantial proportion of HCPs in this study were at least 25% likely to leave their organization and/or profession. Moreover, approximately 15% and 8% of the sample reported certainty in their turnover likelihood for both intentions to leave an organization and profession, respectively. Our qualitative findings expand upon these quantitative results by revealing that most HCPs surveyed were wrestling with the costs and benefits of leaving their healthcare organization and/or profession. Whether a HCP perceived that their work was not worth the toll it was taking or found themself wanting to leave but feeling prevented from doing so for personal or pragmatic reasons, HCPs appeared to be at different spots in a complex internal negotiation process of intending to leave their organization and/or profession. Here, our qualitative findings expanded upon the quantitative turnover likelihood reports by suggesting that reporting a lower likelihood to leave did not necessarily indicate that the HCPs wanted to stay in their current occupation or profession; rather, a low turnover likelihood appeared to indicate that specific to the present moment, the cons of leaving were perceived to outweigh any benefits of leaving, rendering a HCP’s decision to remain employed vulnerable to change. These findings should raise alarm, given their revelation that the continuity of care in the Canadian healthcare system could be unstable. Many HCPs appear to be remaining in a healthcare position post-COVID-19 pandemic where they perceive they are overworked, underappreciated, unsupported, struggling with mental health and well-being, and are poised to leave as soon as a better opportunity arises. This unsettling picture suggests that retention in the healthcare system remains uncertain and the workforce who remains may be suffering psychologically and physically and consequently unable to provide optimal care to patients.

Concerning the influences on turnover intention, both the qualitative and quantitative results point toward the important role of increased years worked (or age), morally distressing events, particularly the witnessing of moral transgressions, burnout, and organizational support. The impact of years worked/age on turnover intention is intuitive as HCPs of older age are more likely to approach retirement and, therefore, more likely to intend to leave their organization and/or profession in the near future. Indeed, many of the qualitative excerpts were one-word responses indicating “retirement” as the qualifying statement for a high turnover likelihood rating.

The strong impact of burnout in our model is consistent with numerous studies, most notably with Nazarov et al.’s [22] findings, where burnout was the sole mental health variable associated with turnover intention, washing out the effects of PTSD, depression, and anxiety in both studies. It is curious that the effect of burnout in the present study additionally washed out the impact of exposure to morally distressing events and trust-violation-related moral injury. The profound impact of burnout in our study appears consistent with Rosen and colleagues’ [40] theoretical continuum, situating burnout as the final stage HCPs face in response to moral stressors in the workplace. It is possible that morally distressing events and morally injurious outcomes, especially trust-violation-related as evidenced in our quantitative and qualitative results, may hasten HCPs down an emotional pathway of increased turnover intention, but it may be the lack of distress, the numbness and depersonalization characteristic of burnout, combined with a lack of perceived organizational support, that appears to be the “tipping point” at which HCPs are poised to leave their organizations and/or professions when a better alternative arises.

Here, it is possible, although unconfirmed, that the relations between morally distressing situations and morally injurious outcomes and turnover intention depend, in part, on the degree of burnout and perceived organizational support experienced either simultaneously or downstream. Indeed, in a study investigating the impact of COVID-19-related work changes and burnout on turnover intention among mental health professionals in the United States, organizational trust and perceived organizational support moderated the positive relation between work changes (e.g., changes to setting or team) and burnout [43]. Moreover, D’Alessandro-Lowe et al. [65] found that organizational support was strongly negatively associated with moral injury among HCPs during the pandemic, including both the shame- and trust-violation subcomponents of moral injury. Preliminary support for this notion comes from our qualitative results, where those who were content to stay described a sense of passion and pride in their work, contrary to the cynicism and detachment from one’s work that characterizes burnout. Moreover, HCPs in the present study who were content to stay were not exempt from the challenging workplace experiences described by others but rather noted supportive leadership as vital to their mental health and sense of safety in their workplace amid workplace challenges. Future work is needed to explore this hypothesis and to better understand the impact of moral stressors, moral injury, and burnout in HCPs’ process of wrestling with the pros and cons of leaving their current employment positions.

Alternatively, however, the profound effect of burnout in our quantitative result may be explained by the specific burnout measure used in this study (BMS) [56], which asks participants to rate experiences of feeling tired, disappointed by people, hopeless, depressed, worthless, helpless, and sickly when thinking about their work. This measure may not adequately capture the definition of burnout discussed more broadly in the literature on turnover intention, which describes burnout as involving emotional exhaustion, depersonalization, and cynicism. Indeed, the items included in the BMS appear relevant for the experience of moral injury, depression, and other types of adverse psychological experiences. Here, we recommend that future research investigating turnover intention among HCPs utilize a burnout measure such as the Maslach Burnout Inventory [66] that may cover the construct more holistically. To follow up on this hypothesis, however, we re-ran the models presented in Table 5 and Table 8 excluding burnout and found the final model to remain composed of years worked (or sex^×^age for professional turnover) and organizational support. This follow-up suggests then that burnout may indeed be important for understanding a driving force for turnover, above and beyond other mental health experiences.

Finally, whereas our quantitative results pointed to perceived organizational support as significantly associated with decreased turnover intention for both intentions to leave an organization and profession, the qualitative accounts provided insight into the inverse relation: a perceived lack of support and sense of betrayal from leadership were depicted in HCPs’ comments on their turnover intention ratings. The important role of organizational support in our study is consistent with a long-standing body of research on the impact of organizational support on job retention and turnover in healthcare [60,67,68,69,70], as well as the literature on burnout, moral distress, and moral injury [22,25,27,65]. Here, our findings and the broader literature point to organizational support as a key opportunity to reinforce the Canadian healthcare workforce and attract individuals to healthcare professions. Indeed, Rosen et al. [40] highlighted that the value of organizing moral distress, moral injury, and burnout on a continuum lies within the opportunity for appropriate intervention. Specifically, while moral stressors may be inevitable in the healthcare context and moral resilience training may help HCPs prepare for and respond to such stressors, moral injury in the healthcare area relates to systemic issues, such as staffing shortages or a lack of appropriate equipment that place HCPs in a position to contribute to or witness compromised patient care, thus disrupting a key value for HCPs. Here, healthcare leadership has the opportunity to support staff by addressing systemic issues that may give rise to moral injury and, in turn, burnout that may lead to turnover [40]. In a narrative review of interpersonal factors that may relate to COVID-19-related moral injury in HCPs, D’Alessandro et al. [17] briefly synthesized recommendations for healthcare organizations, noting the importance of ensuring that efforts to support HCPs are aligned with what HCPs request and need. This begins by proactively and genuinely seeking HCPs’ opinions and perspectives on their experiences and desired support [17]. For more information on how healthcare organizations can best support their staff, please see our research group’s evidence-informed guidelines: https://healthcaresalute-soinsdesantesalute.com/resources/organizational-recommendations/ (accessed on 1 May 2024).

### 4.4. Strengths and Limitations

This research has strengths in the use of both quantitative and qualitative data collection and analysis, which yielded a richer and more nuanced account of turnover intention than either form of data could offer in isolation. Moreover, a generous sample of HCPs was included in the analysis, allowing for the inclusion of a broad range of HCPs’ perspectives and reports. To maximize the sample for the quantitative portion of the study, multiple imputation was rigorously applied to address missing data and provide more accurate parameter estimates for statistical models than other techniques to address missingness (e.g., complete case, mean substitution). Finally, this study also has strengths in indexing both intentions to leave one’s organization and profession.

The findings of this research must, however, be interpreted within the context of methodological limitations. For example, although several hundred HCPs were included in the study, the sample of HCPs may not be representative of those employed in the healthcare sector across the nation. Indeed, our sample appeared primarily composed of nurses who resided in Ontario. As such, it is unclear to what extent the results of this study may be generalizable to the whole of the Canadian HCP field. From a qualitative perspective, our findings are limited by the constraints of qualitative analysis applied to open-field textboxes on an online survey. This data collection method, although useful in capturing data from a broad range of participants, is limited, as follow-up and clarification are not permitted. Indeed, many HCPs offered one-word data excerpts (e.g., “retirement”) that are unclear and difficult to include in thematic analysis. Relatedly, as participants rated and then commented on their likelihood to leave their organization prior to answering the same questions with reference to their profession, many HCPs only provided a comment related to their organization or wrote “same as above” for their response regarding their likelihood to leave their profession. Future research should consider making use of semi-structured interviews with HCPs to better understand their experiences of turnover intention.

## 5. Conclusions

This study was designed to gain a comprehensive understanding of turnover intention among Canadian HCPs during the COVID-19 pandemic. Our results indicated that HCPs faced challenging working conditions created by staffing shortages and high workloads that, in turn, negatively impacted patient care and HCPs’ mental health and well-being. Moreover, HCPs perceived that leaders at multiple levels did not acknowledge the pandemic circumstances HCPs worked through during the pandemic, nor did they offer adequate support. Many HCPs described wrestling with the idea of leaving their organization or profession in some capacity, suggesting that those remaining in a healthcare position perceive they are overworked, underappreciated, unsupported, struggling with mental health issues, and are ready to leave as soon as a better opportunity arises. While morally distressing events and trust-violation-related moral injury may play a role in turnover intention, burnout and organizational support appear to be important targets for retaining a strong healthcare workforce.

## Figures and Tables

**Table 1:** Quantitative Data Collection Survey Instruments.

Construct	Survey	Description
Demographics	N/A	Healthcare providers (HCPs) were asked to provide basic demographic (e.g., age, sex, gender, race, province of residence) and occupational (e.g., profession, years worked, occupational setting, employment status) information.
Turnover Intention	N/A	HCPs were asked to report their current likelihood of leaving both their organization and their profession, separately, on a 5-point scale ranging from 0% to 100% likely. The survey item read: “Given the current situation, please indicate the likelihood that you will leave your organization/employer”. The same wording was used for a second question with reference to leaving one’s profession.
Moral Distress	Measure of Moral Distress—Healthcare Professional (MMD-HP) [23]	The MMD-HP was used to assess exposure to morally stressful events, as per Houle et al.’s [55] recommendation on the most appropriate usage for this scale. Participants read 27 statements about morally distressing events in the healthcare context and were asked to rate each item twice, once for frequency of occurrence and once for degree of distress. Participants rated each item on a scale from “0—Never/None” to “4—Very Frequently/Very Distressing”. Total scores were calculated by summing the products of the frequency and distress scores. Internal consistency in the present sample was α = 0.95 for the frequency scale and α = 0.97 for the distress scale.
Moral Injury	Moral Injury Outcomes Scale (MIOS) [30]	The MIOS was used to assess for moral injury. The MIOS is a 14-item Likert-type scale in which participants are asked to rate their degree of agreement with items on a scale ranging from 0—Strongly Disagree to 4—Strongly Agree. The MIOS has a two-factor structure: shame-related moral injury (sum of items 1, 3, 7, 8, 12, 13, 14) and trust-violation-related outcomes (sum of items 2, 4, 5, 6, 9, 10, 11). Internal consistency in the present sample was α = 0.86 for the shame subscale and 0.78 for the trust-violation subscale.
Burnout	Burnout Measure Short (BMS) [56]	The BMS was used to assess burnout. The BMS is a 10-item scale that asks participants to rate their level of agreement with various aspects of burnout (e.g., helpless, trapped, hopeless) on a scale ranging from 1—Never to 7—Always with respect to their work-related experiences. Total scores were calculated by summing participant responses across items. Internal consistency in the present sample was α = 0.92.
Post-traumatic Stress	Post-Traumatic Checklist for DSM-5 (PCL-5) [57]	The PCL-5 was used to assess post-traumatic stress. Participants read 20 statements and rated their agreement with each statement on a scale from 0 to 4. Total scores were calculated by summing participant responses across the 20 items. A score between 31 and 33 has been considered as indicative of potential PTSD. Internal consistency in the present sample was α = 0.96.
Depression	Depression Anxiety Stress Scale-21 (DASS-21) [58]	The depression subscale of the DASS-21 was used to assess symptoms of depression. The DASS-21 includes 21 items and asks participants to read and rate their degree of agreement with each item on a scale ranging from 0—Never to 3—Almost Always based on the item’s relevance over the past week. Interpretation of depression scores is as follows: 0–9 Normal, 10–13 Mild, 14–20 Moderate, 21–27 Severe, 28+ Extremely Severe. The internal consistency of the depression subscale in the present sample was α = 0.91.
Anxiety	Depression Anxiety Stress Scale-21 (DASS-21) [58]	The anxiety subscale of the DASS-21 was used to assess symptoms of depression. The DASS-21 includes 21 items and asks participants to read and rate their degree of agreement with each item on a scale ranging from 0—Never to 3—Almost Always based on the item’s relevance over the past week. Interpretation of anxiety scores is as follows: 0–7 Normal, 8–9 Mild, 10–14 Moderate, 15–19 Severe, 20+ Extremely Severe. The internal consistency of the anxiety subscale in the present sample was α = 0.84.
Resilience	Brief Resilience Scale (BRS) [59]	The BRS was used to assess resilience, or one’s ability to “bounce back” in the face of stress. Participants read 6 items and rated their degree of agreement with each statement on a scale from 1—Strongly Disagree to 5—Strongly Agree. Total scores were calculated by summing participant responses. The internal consistency in the present sample was α = 0.88.
Organizational Support	Survey of Perceived Organizational Support (SPOS) [60]	The 16-item version of the SPOS was used to assess the degree to which HCPs believed their “organization values their contributions and cares about their well-being” [60] (p. 1). Participants were asked to rate their degree of agreement with 16 statements on a 7-point Likert-type scale ranging from strongly disagree to strongly agree. Total scores were calculated by taking the average of participant responses. The internal consistency in the present sample was α = 0.93.
Social Support	Multidimensional Measure of Social Support (MSPSS) [61]	The MSPSS was used to assess social support across the domains of family, friends, and significant others. The MSPSS is a 12-item scale where participants are asked to rate their degree of agreement with statements regarding social support on a Likert-type scale ranging from very strongly disagree to very strongly agree. Total scores were calculated by taking the average score across items. The internal consistency in the present sample was α = 0.94.

**Table 2:** Sample Characteristics (N = 398).

Variable	Level	Frequency	Percentage
Sex			
	Male	33	8.3
	Female	364	91.5
	Missing	<5	-
Gender			
	Male	31	7.8
	Female	360	90.5
	Gender Diverse	5	1.3
	Missing	<5	-
Ethnicity *			
	African	5	1.3
	Caribbean	6	1.5
	East Asian	9	2.3
	First Nations, Inuit, or Metis	20	5.0
	Latin American	5	1.3
	Middle Eastern	5	1.3
	South Asian	11	2.8
	Southeast Asian	<5	-
	European	244	61.3
	Other	75	18.8
	Missing	22	5.5
Province			
	British Columbia	38	9.5
	Alberta	32	8.0
	Saskatchewan	7	1.8
	Manitoba	23	5.8
	Ontario	248	62.3
	Quebec	6	1.5
	New Brunswick	21	5.3
	Nova Scotia	14	3.5
	Prince Edward Island	<5	-
	Newfoundland/Labrador	<5	-
	Northwest Territories	<5	-
	Yukon	<5	-
	Missing	<5	-
Profession			
	Registered (Practical) Nurse	225	56.5
	Medical Physician	14	3.5
	Respiratory Therapist	6	1.5
	Personal Support Worker	25	6.3
	Occupational Therapist	11	2.8
	Physiotherapist	5	1.3
	Social Worker	32	8.0
	Recreational Therapist	10	2.5
	Other	70	17.6
	Missing	<5	-
Occupational Setting			
	Acute Care Hospital	166	41.7
	Primary Care	21	5.3
	Mental Health Hospital	12	3.0
	Rehabilitation Hospital	<5	-
	Long-term Care or Retirement	99	24.9
	Community or Home Care	31	7.8
	Public Health	22	5.5
	Other	45	11.3
	Missing	<5	-
Employment Status			
	Full-time	285	71.6
	Part-time	84	21.1
	Casual	15	3.8
	Student	<5	-
	Other	10	2.5
	Missing	<5	-
Turnover Intention—Organization			
	0% Likely	83	20.9
	25% Likely	102	25.6
	50% Likely	98	24.6
	75% Likely	55	13.8
	100% Likely	58	14.6
	Missing	<5	-
Turnover Intention—Profession			
	0% Likely	125	31.4
	25% Likely	112	28.1
	50% Likely	90	22.6
	75% Likely	34	8.5
	100% Likely	33	8.3
	Missing	<5	-

Cells with less than 5 counts are suppressed to protect anonymity. * Participants were permitted to select multiple responses.

**Table 3:** Descriptive Statistics for Quantitative Variables.

Construct	Scale	N	Median	*M*	*SD*
Moral Distress	MMD-HP	218	130.50	137.87	97.18
Moral Injury—Shame	MIOS	314	8.00	8.59	5.96
Moral Injury—Trust	MIOS	315	13.00	12.58	5.55
Burnout	BMS	297	44.00	43.68	12.78
Post-traumatic Stress	PCL-5	291	27.00	28.58	18.94
Depression	DASS-21	279	12.00	13.48	9.83
Anxiety	DASS-21	279	8.00	9.41	8.05
Resilience	BRS	265	3.00	3.14	0.85
Social Support	MSPSS	281	5.42	5.23	1.27
Organizational Support	SPOS	379	3.31	3.54	1.43

MMD-HP: Measure of Moral Distress—Healthcare Professional. MIOS: Moral Injury Outcomes Scale. PCL-5: Post-traumatic Checklist for DSM-5. DASS-21: Depression Anxiety Stress Scale-21. BRS: Brief Resilience Scale. MSPSS: Multidimensional Scale of Perceived Social Support. SPOS: Survey of Perceived Organizational Support.

**Table 4:** Simple Ordinal Logistic Regressions: Turnover Intention—Organization.

				OR 95%CI	
	*B*	*SE(B)*	Exp(*B)*	Lower	Upper
Sex	−0.15	0.3232	0.861	0.457	1.623
Age	0.006	0.0077	1.006	0.990	1.021
Sex^×^Age					
Male	0.001	0.01	1.001	0.981	1.234
Female	0.006	0.0078	1.006	0.991	1.021
Years Worked	0.021	0.0081	1.021 *	1.005	1.038
Moral Distress	0.006	0.0013	1.006 **	1.004	1.009
Moral Injury—SR	0.061	0.0168	1.063 **	1.029	1.099
Moral Injury—TVR	0.107	0.0187	1.113 **	1.074	1.155
Burnout	0.087	0.0083	1.091 **	1.074	1.110
Post−Traumatic Stress	0.022	0.0058	1.022 **	1.010	1.034
Depression	0.038	0.0115	1.039 **	1.015	1.062
Anxiety	0.042	0.0134	1.043 **	1.015	1.070
Resilience	−0.093	0.1292	0.911	0.707	1.176
Social Support	−0.135	0.0897	0.874	0.732	1.044
Organizational Support	−0.731	0.075	0.481 **	0.416	0.558

Moral Injury—SR: shame-related subscale of the MIOS. Moral Injury–TVR: trust-violation-related subscale of the MIOS. * Significance at the alpha level *p* = 0.05. ** Significance at the alpha level *p* = 0.01.

**Table 5:** Ordinal Logistic Regression: Organizational Turnover Intention.

	Model 1		Model 2		Model 3		Model 4	
	*B* (SE)	*OR* (95% CI)	*B* (SE)	*OR* (95% CI)	*B* (SE)	*OR* (95% CI)	*B* (SE)	*OR* (95% CI)
Years Worked	0.021 (0.008)	1.021 (1.005, 1.038) *	0.024 (0.008)	1.024 (1.007, 1.041) **	0.019 (0.009)	1.019 (1.002, 1.037) *	0.018 (0.009)	1.018 (1.001, 1.036) *
Moral Distress			0.004 (0.001)	1.004 (1.001, 1.007) **	0.002 (0.001)	1.002 (0.999, 1.005)	0.00 (0.002)	1.00 (0.997, 1.003)
Moral Injury—SR			0.019 (0.019)	1.019 (0.982, 1.059)	0.009 (0.021)	1.009 (0.969, 1.050)	0.005 (0.021)	1.005 (0.964, 1.047)
Moral Injury—TVR			0.081 (0.022)	1.084 (1.039, 1.132) **	0.016 (0.025)	1.016 (0.968, 1.067)	0.006 (0.026)	1.006 (0.956, 1.058)
Burnout					0.088 (0.011)	1.092 (1.069, 1.115) **	0.066 (0.011)	1.068 (1.045, 1.093) **
Post-traumatic Stress					−0.011 (0.009)	0.989 (0.970, 1.008)	−0.006 (0.010)	0.994 (0.975, 1.013)
Depression					−0.009 (0.017)	0.991 (0.959, 1.025)	−0.006 (0.017)	0.994 (0.962, 1.027)
Anxiety					0.004 (0.020)	1.004 (0.967, 1.044)	0.006 (0.021)	1.006 (0.966, 1.049)
Organizational Support							−0.431 (0.092)	0.650 (0.542, 0.779) **
	Nagelkerke *R*^2^ = 0.017		Nagelkerke *R*^2^ = 0.239		Nagelkerke *R*^2^ = 0.279		Nagelkerke *R*^2^ = 0.348	

Moral Injury—SR: shame-related subscale of the MIOS. Moral Injury—TVR: trust-violation-related subscale of the MIOS. * Significance at the alpha level *p* = 0.05. ** Significance at the alpha level *p* = 0.01.

**Table 6:** Final Model: Turnover Intention—Organization.

				OR 95%CI	
	*B*	*SE*(*B*)	Exp(*B*)	Lower	Upper
Years Worked	0.018	0.009	1.018 *	1.001	1.036
Burnout	0.059	0.010	1.061 **	1.041	1.081
Organizational Support	−0.467	0.085	0.627 **	0.530	0.741

* Significance at the alpha level *p* = 0.05. ** Significance at the alpha level *p* = 0.01.

**Table 7:** Simple Ordinal Logistic Regressions: Turnover Intention—Profession.

				OR 95%CI	
	*B*	*SE*(*B*)	Exp(*B*)	Lower	Upper
Sex	−0.362	0.3305	0.696	0.365	1.331
Age	0.018	0.0079	1.018 *	1.002	1.034
Sex^×^Age					
Male	0.008	0.0102	1.008	0.988	1.028
Female	0.019	0.0079	1.019 *	1.004	1.036
Years Worked	0.027	0.0082	1.027 **	1.011	1.044
Moral Distress	0.007	0.0013	1.007 **	1.004	1.010
Moral Injury—SR	0.053	0.0168	1.054 **	1.020	1.090
Moral Injury—TVR	0.105	0.0193	1.111 **	1.069	1.154
Burnout	0.082	0.0084	1.085 **	1.068	1.104
Post-traumatic Stress	0.022	0.0058	1.022 **	1.010	1.034
Depression	0.04	0.0119	1.041 **	1.016	1.066
Anxiety	0.042	0.0136	1.043 **	1.016	1.071
Resilience	−0.134	0.1353	0.875	0.669	1.142
Social Support	−0.152	0.0894	0.859	0.720	1.271
Organizational Support	−0.612	0.0751	0.542 **	0.468	0.628

Moral Injury—SR: shame-related subscale of the MIOS. Moral Injury—TVR: trust-violation-related subscale of the MIOS. * Significance at the alpha level *p* = 0.05. ** Significance at the alpha level *p* = 0.01.

**Table 8:** Ordinal Logistic Regression: Turnover Intention—Profession.

	Model 1		Model 2		Model 3		Model 4	
	*B* (SE)	*OR* (95% CI)	*B* (SE)	*OR* (95% CI)	*B* (SE)	*OR* (95% CI)	*B* (SE)	*OR* (95% CI)
Sex^×^Age								
Male	0.008 (0.010)	1.008 (0.988, 1.028)	0.012 (0.011)	1.012 (0.991, 1.034)	0.019 (0.011)	1.019 (0.998, 1.041)	0.019 (0.011)	1.019 (0.998, 1.041)
Female	0.019 (0.008)	1.019 (1.004, 1.036) *	0.024 (0.008)	1.024 (1.008, 1.041) **	0.028 (0.009)	1.028 (1.011, 1.046) **	0.029 (0.009)	1.029 (1.012, 1.046) **
Moral Distress			0.005 (0.0014)	1.005 (1.002, 1.008) **	0.003 (0.002)	1.003 (1.000, 1.006)	0.001 (0.002)	1.011 (0.998, 1.004)
Moral Injury—SR			0.006 (0.020)	1.006 (0.967, 1.047)	−0.010 (0.023)	0.990 (0.946, 1.037)	−0.01 (0.023)	0.990 (0.946, 1.036)
Moral Injury—TVR			0.082 (0.024)	1.085 (1.035, 1.139) **	0.024 (0.026)	1.024 (0.973, 1.079)	0.017 (0.026)	1.017 (0.967, 1.071)
Burnout					0.082 (0.011)	1.085 (1.063, 1.108) **	0.066 (0.012)	1.068 (1.044, 1.093) **
Post-traumatic Stress					−0.008 (0.011)	0.992 (0.971, 1.014)	−0.005 (0.010)	0.995 (0.975, 1.016)
Depression					−0.002 (0.0205)	0.998 (0.957, 1.040)	−0.002 (0.020)	0.998 (0.959, 1.039)
Anxiety					0.009 (0.020)	1.009 (0.969, 1.049)	0.01 (0.020)	1.01 (0.971, 1.051)
Organizational Support							−0.311 (0.095)	0.733 (0.609, 0.882) **
	Nagelkerke *R*^2^ = 0.019		Nagelkerke *R*^2^ = 0.267		Nagelkerke *R*^2^ = 0.345		Nagelkerke *R*^2^ = 0.361	

Moral Injury—SR: shame-related subscale of the MIOS. Moral Injury—TVR: trust-violation-related subscale of the MIOS. * Significance at the alpha level *p* = 0.05. ** Significance at the alpha level *p* = 0.01.

**Table 9:** Final Model: Turnover Intention—Profession.

				OR 95%CI	
	*B*	*SE(B)*	Exp(*B)*	Lower	Upper
Sex^×^Age	0.018	0.011	1.018	0.997	1.040
Male	0.028	0.008	1.028	1.012	1.045
Female	0.067	0.010	1.069 **	1.049	1.090
Burnout	−0.341	0.088	0.711 **	0.599	0.845
Organizational Support	0.018	0.011	1.018 **	0.997	1.040

** Significance at the alpha level *p* = 0.01.

**Table 10:** Joint Display Table of Quantitative and Qualitative Results with Mixed-Methods Comparison.

Quantitative	Qualitative	Mixed-Methods Comparison
Turnover Intention		
Organization 0% Likely 20.9% 25% Likely 25.4% 50% Likely 24.7% 75% Likely 13.9% 100% Likely 14.6% 78.6% of participants reported being at least 25% likely to leave their current healthcare organization. Profession 0% Likely 31.5% 25% Likely 28.0% 50% Likely 22.7% 75% Likely 8.6% 100% Likely 8.3% 67.5% of participants reported being at least 25% likely to leave their current healthcare profession.	There were no distinct patterns of meaning identified between qualitative responses pertaining to organizational turnover vs. professional turnover. Furthermore, patterns of meaning drawn from the data were not distinguishable across the levels of turnover likelihood. As such, the qualitative data were analyzed as a whole rather than based on subgroups. HCPs who described contentment to stay in their organization or professional highlighted the value of supportive leadership and noted the pride and passion they hold for their work. HCPs described a process of wrestling with the costs and benefits of remaining in their occupation and/or profession. Some HCPs expressed that the toll of the working circumstances outweighed any benefit of remaining in their occupation or profession. Others had a stronger desire to leave yet felt constrained from leaving due to practical or personal reasons. Finally, some HCPs described being on the lookout for a better employment option, such that they were ready to make the move if something else came up.	Convergence and Expansion Both the quantitative and qualitative results speak to the idea that most HCPs surveyed were somewhere on a continuum of intending to leave their organization and/or profession. The qualitative data expand upon the quantified turnover likelihood ratings by suggesting that HCPs’ ratings may differ as HCPs may find themselves at different places of weighing the costs and benefits of leaving their organization/employer. The qualitative findings further expand the quantitative results by suggesting that a HCP who rated a turnover likelihood of 50%, for example, is not necessarily uninterested in leaving but rather may be at a different place of wrestling costs and benefits than someone who rated their turnover intention at 75% or 100%.
Influences on Turnover Intention		
Organization Years worked, moral distress, and trust-violation-related moral injury were each significantly associated with increased odds of turnover intention in the second iteration of the model. The effects of moral distress and trust-violation-related moral injury were, however, washed out by the addition of burnout in the third iteration of the model. The final model indicated that whereas years worked and burnout were associated with increased odds of turnover intention, perceived organizational support was associated with decreased odds of turnover intention. Profession The same pattern of results was found for intention to leave a profession, save for the importance of age-by-sex interaction in place of years worked.	While the qualitative data did not explicitly set out to identify “factors” associated with turnover intention, the themes “Drowning and No One Cares” and “Moral Stressors” provide insight into influences on HCPs’ experiences and perspectives of turnover intention. Drowning and No One Cares HCPs described working in challenging conditions that introduced significant strain on mental health and well-being. These experiences were further complicated by a perceived lack of acknowledgment and support from leadership. Moral Stressors Amid such working conditions, HCPs described a transgression of their value to provide adequate patient care and to be cared for by leaders. Furthermore, many HCPs noted “retirement” in their comments qualifying their turnover likelihood ratings.	Convergence and Expansion The qualitative data provide context to the quantitative findings. The quantitative results demonstrate that HCPs who are more likely to leave are those who perceive low organizational support, those who report high levels of burnout, and those who report an older age. The qualitative findings expand upon these results by painting the picture of challenging working conditions that created situations that violated HCPs’ values and resulted in a perception that leadership did not care about them. Moreover, the qualitative results expand upon the quantitative finding that age is associated with increased turnover likelihood by pointing out that many individuals noted retirement as a reason for qualifying their turnover ratings.

Common domains accounted for in the quantitative and qualitative datasets are tabulated along with a summary of the respective quantitative and qualitative results. Comparisons regarding the degree to which the quantitative and qualitative results converge, diverge, or expand results are noted.

## Data Availability

The data presented in this study are available on request from the corresponding author due to privacy reasons.

## Public Involvement Statement

The knowledge and experiences of research team members with training backgrounds in nursing and clinical psychology guided the development and execution of this study. Healthcare providers who participated in this research contributed to the methods and results via closed- and open-ended survey responses.

## Guidelines and Standards Statement

This manuscript was drafted against the “Journal article reporting standards for qualitative primary, qualitative meta-analytic, and mixed methods research in psychology: The APA Publications and Communications Board task force report” for mixed-methods research.
